# Improving hybrid rice breeding programs via stochastic simulations: number of parents, number of hybrids, tester update, and genomic prediction of hybrid performance

**DOI:** 10.1007/s00122-023-04508-6

**Published:** 2023-12-12

**Authors:** Roberto Fritsche-Neto, Jauhar Ali, Erik Jon De Asis, Mehrzad Allahgholipour, Marlee Rose Labroo

**Affiliations:** 1 International Rice Research Institute (IRRI), Los Banos, Philippines; 2 H. Rouse Caffey Rice Research Station, LSU AgCenter, Rayne, USA; 3 Excellence in Breeding Platform, Consultative Group of International Agricultural Research, Lisbon, Mexico; 4 International Maize and Wheat Improvement Center (CIMMYT), Texcoco, Mexico

## Abstract

**Key message:**

Schemes that use genomic prediction outperform others, updating testers increases hybrid genetic gain, and larger population sizes tend to have higher genetic gain and less depletion of genetic variance

**Abstract:**

One of the most common methods to improve hybrid performance is reciprocal recurrent selection (RRS). Genomic prediction (GP) can be used to increase genetic gain in RRS by reducing cycle length, but it is also possible to use GP to predict single-cross hybrid performance. The impact of the latter method on genetic gain has yet to be previously reported. Therefore, we compared via stochastic simulations various phenotypic and genomics-assisted RRS breeding schemes which used GP to predict hybrid performance rather than reducing cycle length, which allows minimal changes to traditional breeding schemes. We also compared three breeding sizes scenarios that varied the number of genotypes crossed within heterotic pools, the number of genotypes crossed between heterotic pools, the number of hybrids evaluated, and the number of genomic predicted hybrids. Our results demonstrated that schemes that used genomic prediction of hybrid performance outperformed the others for the average interpopulation hybrid population and the best hybrid performance. Furthermore, updating the testers increased hybrid genetic gain with phenotypic RRS. As expected, the largest breeding size tested had the highest rates of genetic improvement and the lowest decrease in additive genetic variance due to the drift. Therefore, this study demonstrates the usefulness of single-cross prediction, which may be easier to implement than rapid-cycling RRS and cyclical updating of testers. We also reiterate that larger population sizes tend to have higher genetic gain and less depletion of genetic variance.

## Introduction

Hybrid breeding via reciprocal recurrent selection (RRS) is thought to be one of the most effective strategies for improving the genetic value of crops with heterosis due to dominance (Cowling et al. 2020). RRS is an emerging breeding strategy for self-pollinating inbred-hybrid crops, such as rice (*Oryza sativa* L.); rice hybrids, which RRS does not always produce, currently outperform the best inbreds by around 30% with appropriate management (Toriyama et al. 2019; Labroo et al. 2021; Lu and Xu, 2010). The main reason to use RRS is that hybrids increasingly take advantage of heterosis due to the dominance over breeding cycles. Heterosis describes a phenomenon in which F_1_ hybrids derived from crosses between heterotic pools with diverging allele frequencies at loci because of the dominance exhibit on average superior performance compared to individuals within heterotic pools, whether inbred or outbred (Matsubara 2020; Cui et al. 2020). Heterosis can also be due to epistasis, but RRS as a strategy primarily targets heterosis due to dominance (Lamkey and Edwards, 1999).

The availability of genomic information can increase the efficacy of RRS. It is well known that genomic selection (GS) can increase the rate of genetic gain in RRS programs by reducing the cycle length (Powell et al., 2020). Genomic information also has a further application in increasing the genetic value of hybrid value if used for genomic prediction of hybrid performance (Kadam et al. 2016). It is typically logistically impossible to make and phenotype all possible hybrid individuals between two pools, so predicting hybrid performance allows identifying individuals likely to be high performing before inter-pool crossing (Hallauer et al. 2010; Kadam et al. 2016). To predict hybrid performance, models are developed from a training population of hybrid phenotypes and maker genotypes (usually from previous cycles of the breeding program) and used (Cui et al. 2020). The hybrid marker profiles are typically deduced from their parent inbreds to save on genotyping costs. Some empirical studies show encouraging results in employing genomic prediction of hybrid performance in hybrid rice (Wang et al. 2017; Matsubara 2020; Cui et al. 2020; Labroo et al. 2021). However, they used static and genetically unbalanced datasets, and the effectiveness of the methods is only based on empirical accuracy.

With genomic prediction, estimation of GCA is also resolvable without testcrossing, so unlike in phenotypic programs, testcrossing is unnecessary. It has been demonstrated that testcrossing is suboptimal in terms of accuracy if used to create a training set for rapid-cycling recurrent genomic selection (Fristche-Neto et al. 2018; Seye et al. 2020). Conversely, the North Carolina design II is the best training set to predict hybrids taken from heterotic pools because, via testcrosses, the tester effect may mask the actual breeding values of the parents, then predictive abilities obtained within the same group but with a different tester can be disappointingly low (Albrecht et al. 2014). However, the impact of genomic prediction of hybrid performance on hybrid value over breeding cycles has not been previously reported to our knowledge (Fristche-Neto et al. 2018; Seye et al. 2020).

The use of RRS as well as genomic information in commercial hybrid rice breeding has not yet been fully adopted. Rapid-cycling reciprocal recurrent genomic selection is a resource-demanding and logistically challenging strategy, and there is a need for strategies to transition to its implementation. Conducting RRS with genomic prediction of hybrid performance without rapid cycling is a logistically attractive transition strategy because it allows infrastructure development to collect, manage, and analyze genomic data without the unforgiving timelines imposed by rapid cycling. Therefore, we evaluate long-term schemes in terms of parameters such as genetic gain, best hybrid performance, genetic variance, heterotic pool divergence, and prediction accuracy by stochastic simulation. We compare breeding scenarios that produce single-cross hybrids phenotypically or with genomic prediction, with or without updating testers in phenotypic cases, and consider three breeding program sizes. Our study aims to provide a transition strategy to use genomic information in RRS schemes to increase genetic gain with minimal changes to phenotypic RRS schemes.

## Materials and methods

Our study compared different rice breeding strategies in a long-term reciprocal recurrent selection program by stochastic simulations in the R package *AlphaSimR* (Gaynor et al. 2021). We considered single crosses that resulted from testcrossing based on phenotypic data, with or without tester updating, vs. single-crosses subset from a North Carolina II design (factorial) predicted from genomic data, including additive or additive + dominance kernels at three different total sizes of the breeding program.

## Historical population and genetic parameters

The historical rice founder population was simulated as 3,000 unique diploid inbred individuals, with 12 chromosome pairs each, using a Markovian Coalescent Simulator (MaCS; (Chen et al. 2009), considering a “GENERIC” species. The number of aggregating segments was defined based on the genome size (cM) described by Li et al. (2008). The “GENERIC” option allows the user to define specific genetic/genomic features in order to represent as much as possible the species in the study.

The target of the simulation was a quantitative trait, such as grain yield (GY). The trait was comprised of 30 QTN per chromosome, totaling 360 QTN. A simulated SNP chip with 83 SNPs per chromosome was used for genotyping, totaling 996 SNPs; SNP and QTN sites were not allowed to overlap. The additive, dominance, and average degree of dominance parameters were defined based on (Li et al. 2008). Each QTN was assigned additive and dominance effects. Total genetic values for each genotype were obtained by summing all additive and dominance effects times the appropriately scaled genotype dosage for all QTN; for details, see Gaynor et al. (2021). Additive effects ($a$) were sampled of a gamma distribution with scale and shape parameters equal to 1 and randomly assigned for each QTN. Similarly, dominance effects ($d$) for each QTN were computed by multiplying the absolute value of its additive effect ($a_{i}$) by locus-specific dominance degree ($\delta_{i}$). Dominance degrees were sampled from a Gaussian distribution with $\delta_{i} \sim N\left( {\mu_{\delta } ,\sigma_{\delta }^{2} } \right)$, where $\mu_{\delta }$ is the average dominance degree equal to 0.22 and $\sigma_{\delta }^{2}$ is the variance of the dominance degrees equal to 0.26. Therefore, there is at least a 33% chance that the delta will be negative (bi-directional dominance deviations) and a 6% chance that it will exceed the unit (overdominance).

The initial mean of the quantitative trait was 0, and its initial total genetic variance was 1. Phenotypic values of each individual were obtained by adding a random error sampled from a Gaussian distribution to its true total genetic value such that initial broad-sense heritability was 0.53 and initial narrow-sense heritability was 0.5; heritability changed over cycles as genetic variances changed. This study did not consider epistasis, even though it may contribute to heterosis in many rice populations (Huang et al. 2016).

## Base population and burn-in phase

**Fig. 1:**
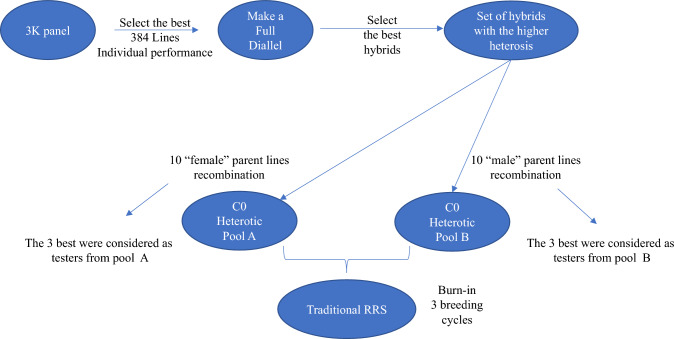
Historical and base populations. 3 K panel: 3,000 inbred lines that formed the historical population and mimicked the germplasm bank; C0: cycle zero; RRS: reciprocal recurrent selection

To obtain the base population, we selected 384 individuals based on their superior phenotypic values from the 3,000 lines of the historical population (Fig. 1). Then, we crossed all the selected lines in silico, obtaining 73,536 single crosses. Later, we selected the ten best hybrids based on their phenotypic performances. From the parents of the best hybrids, we identify the ten best and unique female and male parents to compose the cycle zero (C0) of heterotic pools (HP) A and B, respectively. Furthermore, the parents from each HP of the three best hybrids were considered testers. Next, we simulated three traditional reciprocal recurrent selection (RRS) cycles totaling nearly 20 years of breeding as the burn-in stage. We used breeding size scenario I, the current size used at IRRI, which will be described later. Finally, we obtained the base breeding population as a reference to evaluate the main objective of this study, different breeding schemes, and sizes for rice hybrid reciprocal recurrent selection breeding programs.

## Breeding schemes simulated

### Traditional (TRAD_RRS), drift (DRIFT_RRS), and traditional updating testers every cycle (TRAD_RRS_ UP) reciprocal recurrent selection

**Fig. 2:**
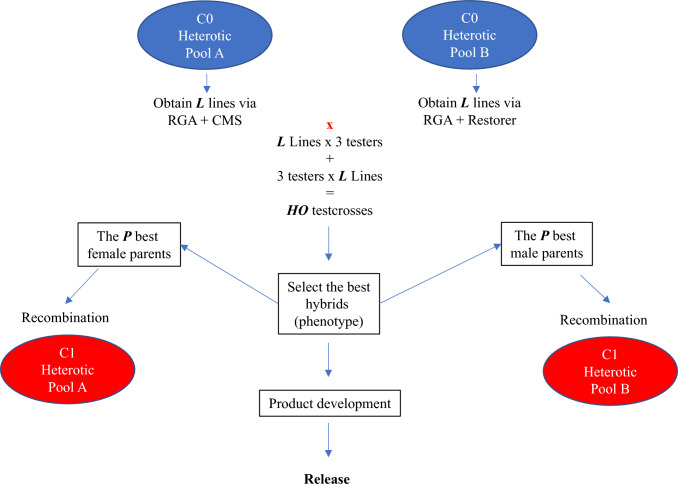
Reciprocal recurrent selection breeding scheme in hybrid rice

The traditional RRS scheme, TRAD_RRS, was conducted following the traditional RRS scheme described by Comstock et al. (1949) adapted to rice (Gilmore 1964) (Fig. 2). First, the selected ***P*** intra-pool parents were randomly mated within each ***HP*** to obtain the ***C1*** recombinant generation. The ***C1*** generation was fully inbred by the rapid generation advanced (RGA) single-seed descent (SSD) method, which accelerates the growth cycle to 90—100 days (Collard et al. 2017). Additionally, we assumed that females and males, respectively, passed through the cytoplasmic male sterile (CMS) conversion and restorer line fixation and its confirmation of fertility restoration in the F_1_ hybrids (Toriyama et al. 2019). Then, a random sample of ***L*** intra-pool lines (Table 1) was obtained from each ***HP*** and crossed with three testers from the first cycle of selection of the reciprocal ***HP***, with the same three testers used for all cycles. Thus, ***HO*** testcrosses (2 pools × ***L*** lines × 3 testers) were obtained and evaluated. After that, we selected the ***P*** best hybrids based on their phenotypic performance, then identified their parents, which the females will belong to ***HP*** A and the males to ***HP*** B. The best phenotypically performed hybrid was also “released” as a variety (Fig. 2). The selected parents were then recycled to restart the cycle.

**Table 1:** Number of lines, hybrids, and parents compared (breeding sizes scenarios) in the stochastic simulations

Method	Traditional			Genomic selection		
Breeding size scenarios	I	II	III	I	II	III
Lines (***L***) used from each ***HP***	64	128	192	64	128	192
Hybrids created in silico (***HI***)	0	0	0	4,096	16,384	36,864
Hybrids actually obtained (***HO***)	384	768	1,152	384	768	1,152
Parents used in recombination (***P***)	10	20	30	10	20	30

The only difference between the TRAD_RSS and DRIFT_RSS is that for DRIFT_RRS a random sample of hybrids was obtained with the same size (***P***), and consequently, the parents were chosen by chance. The DRIFT_RRS is important to detangle/separate the changes in genetic variability due to selection or by genetic drift (small sample size). The latter happens when a small number of parents are used for recombination. It is a well-known phenomenon in the literature (Walsh and Lynch 2018). Finally, in the TRAD_RSS_UP, the three testers of each HP were replaced with improved ones (the three best testcross parents) every breeding cycle.

### Genomic additive (GS_A_RRS) and additive + dominance (GS_AD_RRS) reciprocal recurrent selection

**Fig. 3:**
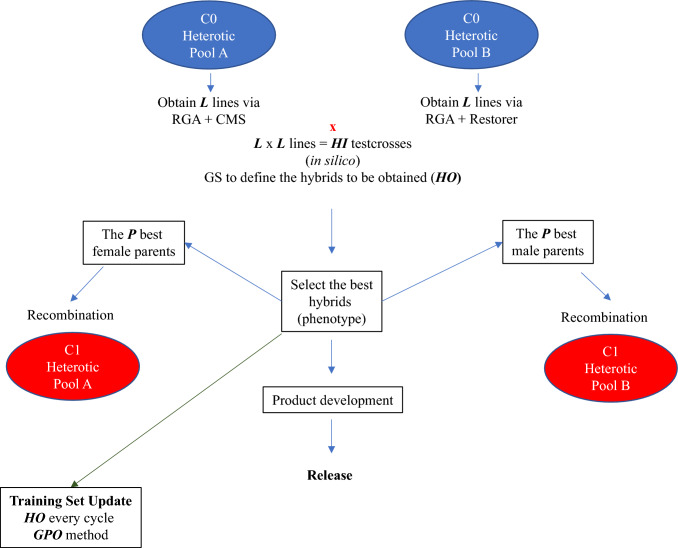
Genomic reciprocal recurrent selection breeding scheme in rice

Overall, the genomic selection schemes followed almost the same framework used in the TRAD_RRS, with few modifications (Fig. 3). The primary difference was that rather than testcrossing, ***L*** inbred lines from each HP were genotyped. All their possible single-cross combinations genotypic values were then predicted in silico, resulting in ***HI*** testcrosses (***L*** × ***L***). The ***HO*** hybrids with the highest total genetic values were advanced to the inter-pool crossing block and phenotypically evaluated (Fristche-neto et al. 2018). As in TRAD_RRS, parents of the next generation were the parents of the ***P*** best-performing hybrids, but the best-performing hybrids were selected by predicted total genetic value rather than phenotype.

To compose the first training set (TS) for GS, we used the phenotypes and marker genotypes of 384 hybrids resulting from the base population after the burn-in stage. Markers’ effects were estimated using the ridge-regression best linear unbiased prediction (RR-BLUP), considering the respective functions in the *AlphaSimR* package for A and A+D (Gaynor et al. 2021). Moreover, we adopted the GPO strategy to update the TS to keep the accuracy at reasonable levels for more breeding cycles (Sabadin et al. 2021). In short, this strategy considers only the last three breeding cycles to compose the training set (Grandparents, Parents, and Offspring). Therefore, we added the newest ***HO*** hybrid data every breeding cycle, removed the oldest one, and maintained only the last three generations in the TS.

To perform the genomic predictions, the AlphasimR package (Gaynor et al. 2021) uses the basic RR-BLUP model for the additive model:

$${\varvec{y}} = {\varvec{WGu}} + \varepsilon$$

where ${\varvec{u}}\sim N\left( {0,{\varvec{I}}\sigma_{u}^{2} } \right)$  is a vector of marker additive effects, ***G*** is the genotype matrix (e.g., {aa,Aa,AA} = {− 1,0,1} for biallelic single nucleotide polymorphisms (SNPs) under an additive model), and ***W*** is the design matrix relating lines to observations (***y***). The BLUP solution for the marker effects can be written as $\hat{u} = \left( {{\varvec{Z}}^{\prime } {\varvec{Z}} + \lambda {\varvec{I}}} \right)^{ - 1} {\varvec{Z}}^{\prime } {\varvec{y}}$, where ***Z*** = ***WG*** and the ridge parameter $\lambda = \sigma_{e}^{2} /\sigma_{u}^{2}$ is the ratio between the residual and marker variances.

Additionally, for the additive-dominance scenario, we used the following model:

$${\varvec{y}} = {\varvec{WGu}} + {\varvec{WTd}} + \varepsilon$$

where ${\varvec{u}}\sim N\left( {0,{\varvec{I}}\sigma_{u}^{2} } \right)$  is a vector of marker additive effects, ${\varvec{d}}\sim N\left( {0,{\varvec{I}}\sigma_{d}^{2} } \right)$  is a vector of marker dominance effects, ***G*** is the genotype matrix (e.g., {aa,Aa,AA} = {− 1,0,1} for biallelic single nucleotide polymorphisms (SNPs) for an additive effects), ***T*** is the genotype matrix (e.g., {aa,Aa,AA} = {0,1,0} for biallelic single nucleotide polymorphisms (SNPs) for dominance effects), and ***W*** is the design matrix relating lines to observations (**y**). The BLUP solutions for the marker effects can be written as $\hat{u} = \left( {{\varvec{Z}}^{\prime } {\varvec{Z}} + \lambda {\varvec{I}}} \right)^{ - 1} {\varvec{Z}}^{\prime } {\varvec{y}}$ and $\hat{d} = \left( {{\varvec{Z}}_{{\varvec{d}}}^{\prime } {\varvec{Z}}_{{\varvec{d}}} + {\varvec{\lambda}}_{{\varvec{d}}} {\varvec{I}}} \right)^{ - 1} {\varvec{Z}}_{{\varvec{d}}}^{\prime } {\varvec{y}}$, where ***Z*** = ***WG*,** and the ridge parameter $\lambda = \sigma_{e}^{2} /\sigma_{u}^{2}$ is the ratio between the residual and marker additive variances. Finally, ***Z***_***d***_ = ***WT***, and the ridge parameter ${\varvec{\lambda}}_{{\varvec{d}}} = \sigma_{e}^{2} /\sigma_{d}^{2}$ is the ratio between the residual and marker dominance variances.

## Breeding scenarios sizes

We also compared different breeding sizes for the most critical stages of the reciprocal selection schemes, such as the number of lines (***L***) sample from each heterotic pool, hybrids obtained in silico (***HI***), and field evaluated/obtained (***HO***), and finally, the number of parents used for recombination within each heterotic pool (Table 1). Therefore, in the end, we have six different breeding frameworks, one benchmark (DRIFT_RSS), and two modeling variations for the GS schemes, GS_A_RSS and GS_AD_RSS. Besides it would be interesting in terms of theory, we avoided simulating unrealistic breeding scenarios, at least for our budget conditions. The main bottleneck to work on the breeding sizes is not the genotyping process but the male sterility and recovery systems/pipelines, which demand a tremendous amount of labor, and thus, defining the maximum number of lines that can be managed per year.

## Comparing breeding schemes

Considering that all compared methods used almost the same framework, there were no differences in cycle length among them. Therefore, we measured the average true genetic value of the ***HO*** hybrids, the true genetic value of the best hybrid, the true additive genetic variance within the heterotic pool, the prediction accuracy, and the divergence between the HP over the breeding cycles. The prediction accuracy was calculated as a Pearson’s correlation between hybrid true genetic values and the hybrid genomic estimated total genetic value. The selection accuracy was computed for the TRAD_RSS scheme as the correlation between hybrid phenotypic value and hybrid true total genetic value. Conversely, for the DRIFT_RRS, it was considered zero because the selection was made by chance. In its turn, the divergence was estimated by the fixation index (*F*_*ST*_) (Luo et al. 2019).

Each strategy was simulated for 20 breeding cycles and replicated 100 times within a single population using the *AlphaSimR* package (Gaynor et al. 2021).

## Results

**Fig. 4:**
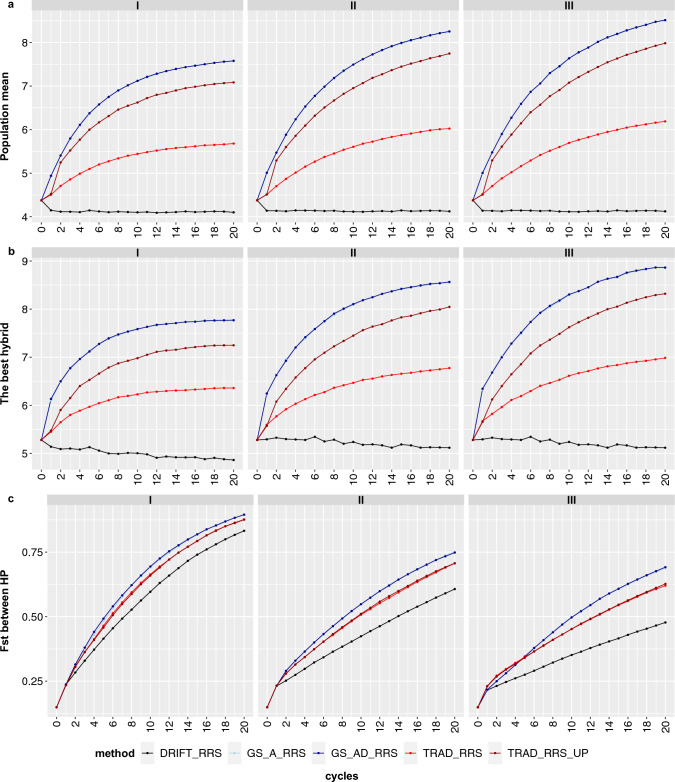
Hybrid population performance mean (**a**), the best genotype (**b**), and fixation index (*F*_*ST*_) between heterotic pools (HP) (**c**) over 20 reciprocal recurrent selection cycles considering three breeding scenarios sizes (I, II, and III). Each colored line represents a selection method

The GS (GS_A_RSS and GS_AD_RRS) methods outperformed the traditional (phenotypic selection, TRAD_RRS) and drift (DRIFT_RRS) for both the average interpopulation hybrid population performance and the best hybrid performance (Fig. 4a, b). Furthermore, updating the testers in TRAD_RRS_UP provides a great increase in response to selection compared to repeated use of the same testers in TRAD_RRS. This trend was regardless of the breeding size, but it was more substantial considering the size III (~ 42%).

**Fig. 5:**
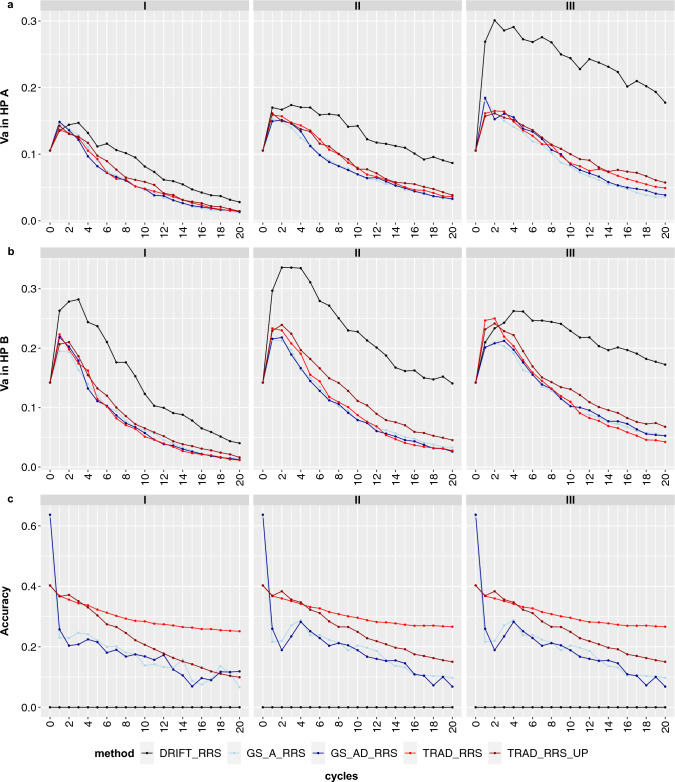
Additive variance in heterotic pools A and B (**a**, **b**) and prediction accuracy (**c**) over 20 reciprocal recurrent selection cycles considering three breeding scenario sizes (I, II, and III). Each colored line represents a selection method

Overall, breeding size scenario III provides higher responses to selection (Fig. 4a) and reduces the losses in additive genetic variance (*V*_a_) by drift (Fig. 5a, b). However, *Va* substantially decreased over the cycles in both heterotic pools regardless of the method.

Regardless of the method, the fixation index (*F*_*ST*_) increased over the cycles, even in the drift scenario, being more substantial in breeding size I and less accentuated considering breeding size III. In the latter, there was a better separation between the *F*_*ST*_ produced by selection or drift (Fig. 4c). Conversely, for all methods, the prediction accuracy significantly declined in the first two cycles (between the TS and first breeding cycles), then presented a steady trend until the 20th cycle.

Comparing the GS methods in terms of modeling, using only additive (GS_A_RRS) or additive + dominance (GS_AD_RRS) to predict hybrid performances, there were no significant differences between them for any parameters studied over the reciprocal recurrent breeding cycles.

Finally, after 20 breeding cycles, using GS and breeding size III rather than TRAD_RRS and size I, it was possible to increase by more than 56% the average interpopulation hybrid population performance and the best hybrid performance. Please note that genomic selection by the A and A + D models has almost overlapped (since the light blue broken line is not visible in many figures).

## Discussion

Simulations have proven to be a powerful tool to assess the main factors in long-term breeding programs because several features can be controlled to make inferences on given genetic parameters over cycles in a fast, inexpensive, and consistent way (Dai et al. 2020). In this study, we successfully used this approach to examine the effectiveness of two main frameworks in employing RRS in hybrid rice breeding, phenotypic and genomic selection (Figs. 4 and 5). Furthermore, we examined three breeding sizes scenarios concerning the number of parents to obtain testcrosses, hybrids obtained and in silico and evaluated them in the field, and the number of parents for recombination within the heterotic pools (Table 1). Moreover, the simulated method mimics the current scenario in the IRRI hybrid rice breeding program (breeding size I) and some possible adjustments in the framework.

Several studies have reported an increase in response to selection in breeding schemes using GS (Gorjanc et al. 2018; Muleta et al. 2019; DoVale et al. 2021; Sabadin et al. 2021). However, to increase the GS-based breeding program efficiency, it is essential to decide how and at which stage to apply this tool (Sabadin et al. 2021). Usually, this increase is due to the shortening of the breeding cycle, which can be logistically challenging. Conversely, in our study, there were no differences in cycle length among breeding frameworks. Instead, by predicting all possible single-cross hybrids and advancing on their genomic predicted total genetic value, it was possible to increase mean hybrid value and maximum hybrid value (Fig. 4a, b). In practice, shortening the cycle by genomic selection on GCA can be used concurrently with genomic prediction of hybrid performance, but starting with use of the latter may be a useful transition strategy for programs moving to use of genomic information.

The first reason single-cross prediction appeared to increase hybrid value was that it enabled regular inter-pool crossing to the most recent generation of the reciprocal HP, rather than using a few testers or even testers from previous breeding cycles. This is apparent because TRAD_RRS_UP had intermediate performance between TRAD_RRS and the GS methods; like TRAD_RRS_UP, the GS methods continuously update both pools rather than only one in TRAD_RRS (Fig. 4a, b). In other words, both parents benefit from population improvement as they are derived from recent breeding cycles in TRAD_RRS_UP and GS methods. The observations of Labroo and Rutkoski (2022) support our findings, as they observed that allowing old parents to be recycled with overlapping generations tended to decrease genetic gain in recurrent selection, and using old testers has a similar effect as using old parents in the RRS context. Although we include the TRAD_RRS_UP scenario to demonstrate this point, in practice, it can be challenging for programs to update testers due to seed limitations, so GS may provide logistical advantages that support regular updating of both HP. Other recent results show that using parents from just one generation back is enough to create a significant “penalty” in the population’s performance (Platten and Fritsche-Neto 2022). Additionally, although we selected testers as the parents of the most recent high-value hybrids, we note that if needed, even a random, more recent tester is likely to produce better hybrids than a selected but older tester.

The second reason single-cross prediction appeared to increase hybrid value was that hybrids were advanced from all possible single-cross combinations on genomic estimated total genetic value rather than testcrossing to three testers. This increases the chances of identifying the best crosses, which is unfeasible with forms of RRS that require a resolvable estimate of GCA (DoVale et al. 2021). Since we did not estimate GCA or SCA in our study and simply recycled and chose testers based on hybrid phenotype or genomic estimated total genetic value (performance) in the previous generation, the decreased performance of the phenotypic methods was partly due to inability to separate the parental GCA effects and SCA effects in the hybrid progeny of the current generation (Cowling et al. 2020). With GS, the prediction of hybrid additive and additive + dominance genetic values is typically equivalent to prediction of parental GCA and SCA, so it was possible to utilize SCA non-randomly in the hybrids via genomic prediction of single-cross performance but not with phenotypic methods (Kadam et al. 2016; Seye et al. 2020). In our study, the use of single-cross prediction also likely indirectly increased the selection differential for GCA, because the rate of increase in hybrid value over cycles was higher than with phenotypic strategies, not just the hybrid value. The use of a reduced NCII design in inter-pool crossing also likely had higher accuracies than a genomics-assisted inter-pool testcrossing design would (Albrecht et al. 2014; Fristche-neto et al. 2018).

Some studies have already shown that information about dominance deviations incorporated in GS models can increase the prediction accuracy (Azevedo et al. 2015; Matias et al. 2018; Dias et al. 2018; Dai et al. 2020), especially when the final product is heterozygous and dominance is present (Dos Santos et al. 2016). Even though only average effects are transmitted over generations, the transmission of average effects via GCA in intra-pool individuals in RRS leads to increased dominance deviations in the inter-pool hybrids over breeding cycles (Falconer and Mackay 1996). In this context, our findings (Fig. 2) show that including dominance effects in the model did not increase accuracy or genetic gain in this population (Technow et al. 2012; Alves et al. 2019b; DoVale et al. 2021). This may be due to the low degree of dominance assumed here for rice yield, which may be lower than in other traits like maize yield (Li et al. 2008; Lin et al. 2020; Labroo et al. 2021). Another possible explanation was described by Reif et al. (2007), where the dominance variance decreases the respective additive variance but increases according to the populations’ divergence. Thus, dominance effects are increasingly absorbed by the population mean or become inseparable from additive effects over breeding cycles (Technow et al. 2014). Therefore, over the breeding cycles, dominance may become more important. Because there was no significant difference in hybrid prediction, including the dominance factor in the GS models did not seem harmful, and in other populations it may be useful. In the presence of dominance, the additive-dominant model is expected to be more accurate, as the additive model falsely assumes that residuals are independent and identically distributed (Duenk et al. 2017). Furthermore, additive-dominant models allow for a more refined assessment of genomic contributions to performance than models that only consider additive effects. However, when we consider only the additive kernel in the models, additive effects, dominance, and residuals are not readily distinguishable (Alves et al. 2019a).

Some possible explanations are that RRS methods not only work based on dominance but also in the other two heterosis components, reorganizing the alleles to maximize and explore divergence and complementary effects (Hallauer et al. 2010). Also, the dominance effect is small in rice compared to other crops, such as maize (DoVale et al. 2021). Finally, the allele substitution effect $\alpha = a + \left( {q - p} \right)d$, when $p \ne q$ and $d \ne 0$, partially captures the dominance effects (Falconer and Mackay 1996). Therefore, the additive model captures, at least in part, the dominance variance. However, the inclusion of dominance in the GS model does not “hurt”, and, in specific cases and germplasm, it might be positive.

This study used an SNP chip containing 996 markers to simulate the SNP panel optimized for the IRRI-irrigated breeding program (Arbelaez et al. 2019). Low-density SNP panels are attractive for GS due to the lower price per sample (Cerioli et al. 2022). In this context, our results showed that even using a low-density marker set, it is possible to keep the accuracy at levels that provide genetic gain over many breeding cycles, however, there is a need to update the TS properly (Sabadin et al. 2021). Furthermore, as described by Sabadin et al. (2021), the GPO updating method promotes more accurate estimates of LD between markers and QTL since, in long-term breeding schemes, recombination between marker and QTL causes an LD decrease. In contrast, selection and drift generate new LD or tighten the LD between closely linked loci. We did not consider the cost of genotyping in comparing our breeding schemes because, for the IRRI program, the cost is negligible, and this will vary depending on institutions.

As expected and well described in the literature, the bigger the population, the bigger the genetic gains. Hence, breeding size III provides higher responses to selection (Fig. 4a). Also, size III was slightly better than others in preserving V_a_, although selection intensity differed among the three breeding sizes (Fig. 5a, b). Any breeding method, mainly those based on GS, will vanish the genetic variability faster than in seen traditional ways. So, it has some main consequences: i) Breeders can reach the plateau of the genetic gains in a population much faster using GS-based methods; ii) After that, one can select a new set of parents and start a new breeding population or even introduce a new genetic *V*_a_. Therefore, there is no case of “the population of the breeder’s life”, in other words, the need to create a population to support the selection for 20–30 years of breeding; iii) As the selection will be very intense, drift must be minimized to guarantee that the losses in genetic variability will be driven by selection.

In this context, it is well known that smaller effective population sizes (***Ne***) increase the effect caused by the drift (Hartl and Clark 2006), reducing genetic variance in long-term breeding cycles. Consequently, it may create a plateau of faster selection response due to the inbreeding effect. Also, the fixation of the alleles in the heterotic groups becomes partly due to random factors (Gerke et al. 2015; DoVale et al. 2021). Reducing inbreeding can be done by increasing the number of parents (breeding sizes) or conserving population genetic diversity via optimal parental contribution (Cowling et al. 2020). Programs considering increasing the size of the breeding population to increase genetic gain or preserve genetic variance should carefully consider whether this intervention is cost-effective. Although some programs may be able to increase the number of entries evaluated at very little cost (e.g., when fixed costs are high and variable costs are low), for others, it may be quite expensive, but this depends on the situation.

Finally, after the horizon of 20 breeding cycles, using GS and breeding size III rather than the phenotypic selection and size I, it is possible to increase by more than 56% the average interpopulation hybrid population performance and the best hybrid performance. Thereby showing the importance of combining the appropriate method of selection, having a dynamic pairwise and updating system of testing using the possibility of creating all possible testcrosses in silico, and the effective population sizes to evaluate and recombine parents to avoid genetic drift and better conserve the genetic variation.

## Data Availability

The datasets and scripts used for this study can be found in the supplementary material.
